# Supplementary material for: From sequence to enzyme mechanism using multi-label machine learning
Source: BMC Bioinformatics. 2014 May 19;15:150. doi: 10.1186/1471-2105-15-150 (PMC4229970; doi:10.1186/1471-2105-15-150)
Supplement: Additional file 2 — Java code of ml2db. Additional file ml2db_code.tar.gz contains the Java source code to run the multi-label machine learning experiments and save the results to database. The code’s Javadoc is included. [file 1471-2105-15-150-S2.zip › additional file 2/ml2db/utils/doc/cern/jet/random/engine/RandomEngine.html]

RandomEngine


JavaScript is disabled on your browser.


- Overview
- Package
- Class
- Use
- Tree
- Deprecated
- Index
- Help

- Prev Class
- Next Class

- Frames
- No Frames

- All Classes

- Summary:
- Nested |
- Field |
- Constr |
- Method

- Detail:
- Field |
- Constr |
- Method


cern.jet.random.engine

## Class RandomEngine

- java.lang.Object
- - cern.colt.PersistentObject
  - - edu.cornell.lassp.houle.RngPack.RandomElement
    - - edu.cornell.lassp.houle.RngPack.RandomSeedable
      - - cern.jet.random.engine.RandomEngine

- All Implemented Interfaces:
  :   DoubleFunction, IntFunction, java.io.Serializable, java.lang.Cloneable

  Direct Known Subclasses:
  :   MersenneTwister

  ---

    

  ```
  public abstract class RandomEngine
  extends RandomSeedable
  implements DoubleFunction, IntFunction
  ```

  Abstract base class for uniform pseudo-random number generating engines.

  Most probability distributions are obtained by using a **uniform**
  pseudo-random number generation engine (derived from this class or
  `RandomElement`) followed by a
  transformation to the desired distribution. Thus, subclasses of this class
  are at the core of computational statistics, simulations, Monte Carlo
  methods, etc.

  Subclasses produce uniformly distributed int's and long's
  in the closed intervals [Integer.MIN\_VALUE,Integer.MAX\_VALUE] and
  [Long.MIN\_VALUE,Long.MAX\_VALUE], respectively, as well as
  float's and double's in the open unit intervals
  (0.0f,1.0f) and (0.0,1.0), respectively.

  Subclasses need to override one single method only: nextInt(). All
  other methods generating different data types or ranges are usually layered
  upon nextInt(). long's are formed by concatenating two 32
  bit int's. float's are formed by dividing the interval
  [0.0f,1.0f] into 2^32^ sub intervals, then randomly
  choosing one subinterval. double's are formed by dividing the
  interval [0.0,1.0] into 2^64^ sub intervals, then randomly
  choosing one subinterval.

  Note that this implementation is **not synchronized**.

  Version:
  :   1.0, 09/24/99

  Author:
  :   wolfgang.hoschek@cern.ch

  See Also:
  :   `MersenneTwister`,
      `Ranmar`,
      `Ranlux`,
      `Ranecu`,
      `Random`,
      Serialized Form

- - ### Method Summary

    Methods

    | Modifier and Type | Method and Description |
    | --- | --- |
    | `double` | `apply(double dummy)` Equivalent to raw(). |
    | `int` | `apply(int dummy)` Equivalent to nextInt(). |
    | `static RandomElement` | `makeDefault()` Constructs and returns a new uniform random number engine seeded with the current time. |
    | `double` | `nextDouble()` Returns a 64 bit uniformly distributed random number in the open unit interval `(0.0,1.0)` (excluding 0.0 and 1.0). |
    | `float` | `nextFloat()` Returns a 32 bit uniformly distributed random number in the open unit interval `(0.0f,1.0f)` (excluding 0.0f and 1.0f). |
    | `abstract int` | `nextInt()` Returns a 32 bit uniformly distributed random number in the closed interval [Integer.MIN\_VALUE,Integer.MAX\_VALUE] (including Integer.MIN\_VALUE and Integer.MAX\_VALUE); |
    | `long` | `nextLong()` Returns a 64 bit uniformly distributed random number in the closed interval [Long.MIN\_VALUE,Long.MAX\_VALUE] (including Long.MIN\_VALUE and Long.MAX\_VALUE). |
    | `double` | `raw()` Returns a 32 bit uniformly distributed random number in the open unit interval `(0.0,1.0)` (excluding 0.0 and 1.0). |

    - ### Methods inherited from class edu.cornell.lassp.houle.RngPack.RandomSeedable

      `ClockSeed, ClockSeed`
    - ### Methods inherited from class edu.cornell.lassp.houle.RngPack.RandomElement

      `choose, choose, gaussian, gaussian, powlaw, raw, raw, uniform`
    - ### Methods inherited from class cern.colt.PersistentObject

      `clone`
    - ### Methods inherited from class java.lang.Object

      `equals, getClass, hashCode, notify, notifyAll, toString, wait, wait, wait`

- - ### Method Detail


    - #### makeDefault

      ```
      public static RandomElement makeDefault()
      ```

      Constructs and returns a new uniform random number engine seeded with the
      current time. Currently this is
      `MersenneTwister`.


    - #### apply

      ```
      public double apply(double dummy)
      ```

      Equivalent to raw(). This has the effect that random engines can
      now be used as function objects, returning a random number upon function
      evaluation.

      **Specified by:**
      :   `apply` in interface `DoubleFunction`

      Parameters:
      :   `dummy` - argument passed to the function.

      Returns:
      :   the result of the function.


    - #### apply

      ```
      public int apply(int dummy)
      ```

      Equivalent to nextInt(). This has the effect that random engines
      can now be used as function objects, returning a random number upon
      function evaluation.

      **Specified by:**
      :   `apply` in interface `IntFunction`

      Parameters:
      :   `dummy` - argument passed to the function.

      Returns:
      :   the result of the function.


    - #### nextDouble

      ```
      public double nextDouble()
      ```

      Returns a 64 bit uniformly distributed random number in the open unit
      interval `(0.0,1.0)` (excluding 0.0 and 1.0).


    - #### nextFloat

      ```
      public float nextFloat()
      ```

      Returns a 32 bit uniformly distributed random number in the open unit
      interval `(0.0f,1.0f)` (excluding 0.0f and 1.0f).


    - #### nextInt

      ```
      public abstract int nextInt()
      ```

      Returns a 32 bit uniformly distributed random number in the closed
      interval [Integer.MIN\_VALUE,Integer.MAX\_VALUE] (including
      Integer.MIN\_VALUE and Integer.MAX\_VALUE);


    - #### nextLong

      ```
      public long nextLong()
      ```

      Returns a 64 bit uniformly distributed random number in the closed
      interval [Long.MIN\_VALUE,Long.MAX\_VALUE] (including
      Long.MIN\_VALUE and Long.MAX\_VALUE).


    - #### raw

      ```
      public double raw()
      ```

      Returns a 32 bit uniformly distributed random number in the open unit
      interval `(0.0,1.0)` (excluding 0.0 and 1.0).

      **Specified by:**
      :   `raw` in class `RandomElement`

      Returns:
      :   a random double in the range (0,1).

      See Also:
      :   `RandomJava`


- Overview
- Package
- Class
- Use
- Tree
- Deprecated
- Index
- Help

- Prev Class
- Next Class

- Frames
- No Frames

- All Classes

- Summary:
- Nested |
- Field |
- Constr |
- Method

- Detail:
- Field |
- Constr |
- Method
